# Liensinine Inhibits Beige Adipocytes Recovering to white Adipocytes through Blocking Mitophagy Flux In Vitro and In Vivo

**DOI:** 10.3390/nu11071640

**Published:** 2019-07-18

**Authors:** Siyu Xie, Yuan Li, Wendi Teng, Min Du, Yixuan Li, Baoguo Sun

**Affiliations:** 1 Beijing Advanced Innovation Center for Food Nutrition and Human Health, College of Food Science & Nutritional Engineering, China Agricultural University, Beijing100083, China; 2 Department of Animal Sciences, Washington State University, Pullman, Washington, WA 99164, USA; 3 Key Laboratory of Functional Dairy, Co-constructed by ministry of Education and Beijing Municipality, College of Food Science & Nutritional Engineering, China Agricultural University, Beijing100083, China; 4 Beijing Technology and Business University, Beijing100083, China

**Keywords:** liensinine, beige adipocyte, obesity, mitophagy, cathepsin protein

## Abstract

Promoting white-to-beige adipocyte transition is a promising approach for obesity treatment. Although Liensinine (Lie), a kind of isoquinoline alkaloid, has been reported to affect white-to-beige adipocyte transition, its effects on inhibiting beige adipocytes recovering to white adipocytes and maintaining the characteristics of beige adipocyte remain unclear. Therefore, we explored the effects and underlying mechanism of Lie on beige adipocyte maintenance in vitro and in vivo. Here, we first demonstrated that after white adipocytes turned to beige adipocytes by rosiglitazone (Rosi) stimuli, beige adipocytes gradually lost their characteristics and returned to white adipocytes again once Rosi was withdrawn. We found that Lie retained high levels of uncoupling protein 1 (UCP1) and mitochondrial oxidative phosphorylation complex I, II, III, IV and V (COX I–V), oxygen consumption rate (OCR) after Rosi withdrawal. In addition, after Rosi withdrawal, the beige-to-white adipocyte transition was coupled to mitophagy, while Lie inhibited mitophagy flux by promoting the accumulation of pro-cathepsin B (pro-CTSB), pro-cathepsin D (pro-CTSD) and pro-cathepsin L (pro-CTSL), ultimately maintaining the beige adipocytes characteristics in vitro. Moreover, through blocking mitophagy flux, Lie significantly retained the molecular characteristics of beige adipocyte, reduced body weight gain rate and enhanced energy expenditure after stimuli withdrawal in vivo. Together, our data showed that Lie inhibited lysosomal cathepsin activity by promoting the accumulation of pro-CTSB, pro-CTSD and pro-CTSL, which subsequently inhibited mitophagy flux, and ultimately inhibited the beige adipocytes recovering to white adipocytes and maintained the characteristics of beige adipocyte after stimuli withdrawal. In conclusion, by blocking lysosome-mediated mitophagy, Lie inhibits beige adipocytes recovering to white adipocytes and may be a potential candidate for preventing high fat diet induced obesity.

## 1. Introduction

Obesity is strongly associated with excessive white adipocytes, and increases the risk of metabolic syndromes or disorders including diabetes, cardiovascular diseases, and multiple types of cancer [1,2,3]. Although clinical care and various medical studies have been made, approximately 28 million patients worldwide die from obesity each year [3]. The mortality and morbidity due to obesity are very high because of its close association with genetic factors, lack of physical activity, and other factors [4,5]. Adipocytes play crucial roles in the development of obesity, with white adipocytes functioning as an energy storage organ, and brown adipocytes and beige adipocytes as an energy consumption organ [6]. With an increase in calorie intake and a decrease in caloric expenditure, excessive white adipocytes store too many fatty acids, thereby resulting in obesity. Increased active brown adipocytes and beige adipocytes can promote energy expenditure, and so may be potential targets for treating metabolic disorders and obesity [6,7,8]. Brown adipocytes constitutively express high levels of uncoupling protein 1 (UCP1) and increase mitochondria mass [9]. However, when white adipocytes respond to chronic cold exposure, β3-adrenergic receptor agonist CL316,243 (β3-AR agonist) or rosiglitazone (Rosi), they transform to beige adipocytes [6]. White-to-beige adipocyte transition is coupled to a UCP1 expression increase and mitochondria mass biogenesis. UCP1 resides in the mitochondrial inner membrane and plays an essential role in dissipating proton gradient in adipocyte tissue to produce heat instead of Adenosine Triphosphate (ATP) production, improving metabolic rates and increasing energy production [10,11,12,13]. The half-life of UCP1 ranges from 1 day to 3.7 days in different studies [14,15,16,17]. In 3T3-L1 adipocyte, forced UCP1 expression can decrease triglycerides synthesis by 36% [18]. UCP1 can protect mice from high fat diet- (HFD)-induced obesity through improving metabolic rate in mice [12]. High mitochondrial mass indicates the upshift of oxidative phosphorylation (OXPHOS) protein level and high oxygen consumption rate (OCR) [19]. High OXPHOS capacity is critical to beige adipocytes [20]. High OCR has been commonly used to evaluate the positive effects of regulators on white-to-beige conversion [21]. By promoting white-to-beige transition, β3-AR agonist helped HFD-induced obesity mice improve their resting metabolic rate. Though weight loss programs were successful, a great deal of the lost weight was regained after stopping treatment with β3-AR agonist. As beige adipocyte tended to translate into white adipocyte, body weight was regained [22]. The beige-to-white adipocyte transition is tightly coupled to thermogenic capacity decrease and mitochondria degradation. By exploring the mechanism of regulating mitochondria degradation in this situation, the authors found that microphthalmia-associated transcription factor, which modulates mitophagy related protein expression levels, was increased on initiation of the beige-to-white transition [22].

Mitophagy (mitochondria-specific autophagy), a conserved lysosome-dependent process, is in charge of degrading mitochondria. It plays a vital role in cellular homeostasis against various human diseases. It also has a close relationship with obesity [23]. Beige adipocytes transform to white adipocytes through a Parkin-dependent mitophagy [24,25]. A reduction in the mitochondrial membrane potential leads to recruitment of Parkin (an E3 ubiquitin ligase that ubiquitinates outer mitochondrial proteins) on the outer mitochondrial membrane, subsequently p62 (selective autophagy adaptor protein) links the ubiquitinated mitochondrial proteins to LC3 (microtubule-associated protein 1 light chain 3), leading to the formation of autophagosomes that target damaged mitochondria [26,27]. Finally, lysosomal proteases are activated to degrade cellular organelles like mitochondria [28,29]. Blocking mitophagy can prolong the maintenance of beige adipocytes, which exacerbated high fat diet (HFD)-induced obesity [22,30]. 

There are very few studies about using plant-derived natural compounds to retain the characteristics of beige adipocytes after stimuli withdrawal. Therefore, developing a novel natural compound to maintain functional beige adipocytes has an important clinical significance for treating obesity. Liensinine (Lie) is a major isoquinoline alkaloid derived from the seed embryo of *Nelumbo nucifera Gaertn* [31]. There is no study about the role of Lie in maintaining beige adipocytes at all. This study was first to assess the effects of Lie on maintaining beige adipocytes and investigate the hypothesis whether Lie could inhibit the beige adipocytes recovering to white adipocytes by blocking mitophagy after stimuli withdrawal both in vitro and in vivo.

## 2. Materials and Methods 

### 2.1. Cell Culture, Differentiation and Establishment of Beige Adipocytes Model

At 37 °C in a humidified atmosphere containing 5% CO_2_, 3T3-L1 mouse embryo fibroblasts (American Type Culture Collection, Manassas, VA, USA) were cultured in six-well plates at a density of 2 × 10^5^ in Dulbecco’s modified Eagle’s medium (DMEM) with 10% fetal bovine serum (FBS, Gibco Life Technologies, Gaithersburg, MD, USA) and grown to confluence. Then the cells were differentiated using DMEM with 10% FBS, 0.25 μM dexamethasone, 2 μg·mL^−1^ insulin and 0.5 mM 3-isobutyl-1-methylxanthine (Sigma, St. Louis, MO, USA) for 3 days. After 3 days, dexamethasone and 3-isobutyl-1-methylxanthine were removed, and incubation with insulin was continued for another 5 days until the cells were fully differentiated into white adipocytes. In order to obtain the model of beige adipocyte, the white adipocytes were treated with 10 μM rosiglitazone (Rosi, Sigma, St. Louis, MI, USA) for 2 days [32,33,34].

### 2.2. Cell Viability Assay

3T3-L1 mouse embryo fibroblasts were seeded into 96-well plates at density of 1 × 10^4^ cells/well and grown to confluence. Then the cells were differentiated using DMEM with 10% FBS, 0.25 μM dexamethasone, 2 μg·mL^−1^ insulin and 0.5 mM 3-isobutyl-1-methylxanthine for 3 days. After 3 days, dexamethasone and 3-isobutyl-1-methylxanthine were removed, and incubation with insulin was continued for another 5 days until the cells were fully differentiated into white adipocytes. White adipocytes were treated with 10 μM Rosi for 2 days to obtain the model of beige adipocyte. After Rosi withdrawal, beige adipocytes were treated with Lie at different concentrations (0, 10, 20, 40 and 60 μM) for 5 days, respectively. According to manufacturer’s instruction, the cells were treated with CCK8 reagent and the absorbance was measured at 450 nm using a microplate reader (Spectra Max, Molecular Devices, MI, USA) [35].

### 2.3. Western Blot Analysis

Cells were lysed in Western-IP lysis buffer (Sigma, USA) at 4 °C. Totally 30 μg of proteins from cell lysates were separated by 12.5% sodium dodecyl sulfate polyacrylamide gel electrophoresis (SDS-PAGE), and transferred onto a polyvinylidene fluoride (PVDF) membrane (Amersham Pharmacia Biotech., Little Chalfont, UK). The membranes were incubated at 4 °C overnight with following primary antibodies: rabbit anti-light chain 3B (LC3) (1:1000, Abcam, Cambridge, UK), anti-p62 (1:1000, Abcam, Cambridge, UK), anti-uncoupling protein 1 (UCP1) (1:1000, Cell Signaling Technology, Beverly, MA, USA) and anti-MitoProfile total OXPHOS rodent antibody cocktail (1:1000, Abcam, Cambridge, UK), anti-Cathepsin B (1:1000, CST, Beverly, MA, USA), anti-Cathepsin D (1:1000, CST, Beverly, MA, USA), anti-Cathepsin L (1:1000, Abcam, Cambridge, UK) and mouse anti-β-actin (1:1000, Sigma, St. Louis, MO, USA). Subsequently, the membranes were subjected to corresponding secondary antibodies [36]. 

### 2.4. Mitochondrial Respiration Study 

Mitochondrial respiration activity analyses were performed using a Seahorse Bioscience XF96 Analyzer (Seahorse Bioscience Inc., North Billerica , MA, USA), which enables real-time simultaneous measurement of oxygen consumption rate. Following basal respiration, oligomycin (1 μM) was injected by automatic pneumatic injection. Sequentially, FCCP (carbonyl cyanide 4 (trifluoromethoxy) phenylhydrazone) (0.75 μM) was injected. Finally, a cocktail of rotenone (1 μM) and antimycin A (1 μM) were injected as described elsewhere [37].

### 2.5. Immunostaining

Immunostaining was performed as described elsewhere, particularly in [19]. The following primary antibodies:anti-Lamp2 (1:100, Proteintech, Beijing, China), anti-LC3 (1:100, CST, Beverly, MA, USA), anti-p62 (1:100, CST, Beverly, MA, USA), anti-Cathepsin B (CTSB) (1:100, CST, Beverly, MA, USA) and anti-Cathepsin D (CTSD) (1:100, Proteintech, Beijing , China) and anti-Parkin (1:100, Proteintech, Beijing, China) and anti-COX IV (1:100, Proteintech, Beijing, China).

### 2.6. Animal Preparation and Experimental Groups

Male C57BL/6J mice (4 weeks old), purchased from Vital River Laboratory (Beijing Vital River Laboratory Animal Technology Co., Ltd., Beijing, China), were maintained at a constant temperature with a 12-h light and 12-h dark cycle. The animal experiments were approved by the Ethics Committee of China Agricultural University (No. AW01049102-4). All mice were fed standard chow diet (12450b, Research Diets, NJ, USA, 10% kcal fat content) and high fat diet (HFD; D12492, Research Diets, NJ, USA, 60% kcal fat content) with free access to food and water and body weights of these mice were recorded weekly. HFD-induced obesity mice were intraperitoneally (i.p.) treated with ß3-AR agonist CL316,243 (Sigma, St. Louis, MO, USA) at a dose of 1 mg·kg^−1^ for seven consecutive days. After the withdrawal of ß3-AR agonist, mice were divided into three groups as follows: HFD with saline (15 days, i.p.), HFD with 60 mg·kg^−1^ CQ (Sigma, St. Louis, MO, USA; 15 days, i.p.) and HFD with 60 mg·kg^−1^ Lie (Yuanye, Shanghai, China; 15 days, i.p.). 

### 2.7. Histology and Immunohistochemistry

Hematoxylin and eosin staining (H&E) and immunohistochemistry assay were conducted as per previous methods in [31]. The tissue specimens were incubated with the following primary antibodies: rabbit anti-light chain 3 (LC3) (1:1000, Abcam, Cambridge, UK), anti-p62 (1:1000, Abcam, Cambridge, UK), anti-uncoupling protein 1 (UCP1) (1:1000, Abcam, Cambridge, UK) and anti-Cathepsin B (1:200, CST, Beverly, MA, USA).

### 2.8. Biochemical Analyses of Serum

Serum total cholesterol (mg·dL^−1^), serum triglycerides (mg·dL^−1^), serum low-density-lipoprotein cholesterol (mg·dL^−1^) and serum high-density-lipoprotein cholesterol (mg·dL^−1^) were analyzed using biochemical assay kits (Nanjing Jiancheng Bioengineering Institute, Nanjing, China). 

### 2.9. Metabolic Assessment

Oxygen consumption, carbon dioxide production, energy expenditure and spontaneous activity were assessed by mouse Comprehensive Laboratory Animal Monitoring System (CLAMS) metabolic cages. Body fat distribution and images were measured by MesoMR23-060H-I imaging instrument (Shanghai Niumag Corporation, Shanghai, China).

### 2.10. Statistical Analysis

All data were presented as mean ± SEM (M ± SEM) and analyzed with ANOVA using SPSS version 20 (SPSS Inc., Chicago, IL, USA). The statistically significant difference was considered at *p* < 0.05.

## 3. Results

### 3.1. Lie Inhibited the Beige Adipocytes Recovering to White Adipocytes and Retained Their Characteristics after Rosi Withdrawal In Vitro 

**Figure 1:**
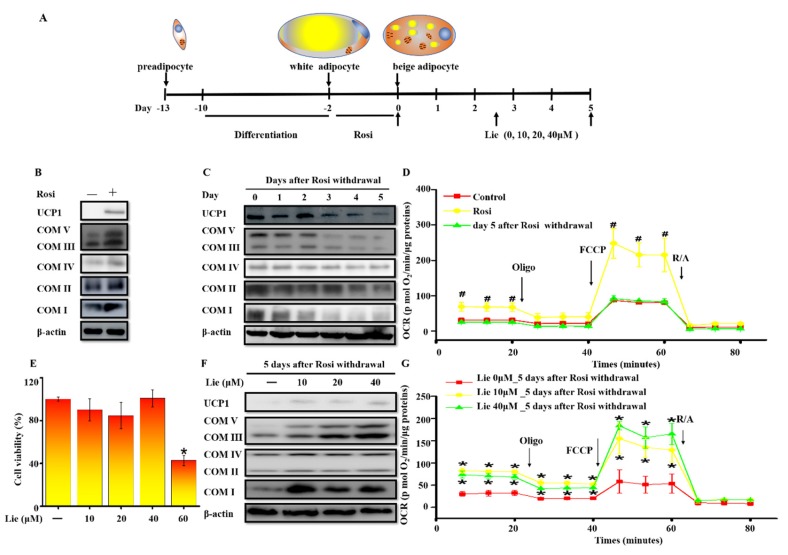
Liensinine (Lie) inhibited the beige adipocytes recovering to white adipocytes and retained their characteristics after rosiglitazone (Rosi) withdrawal in vitro. (**A**) Schematic illustration about in vitro experiments. Preadipocytes were differentiated into white adipocytes. Beige adipocytes were induced by treating white adipocytes with 10 μM Rosi for 2 days. After 10μM of Rosi withdrawal, beige adipocytes were exposed to various concentrations (0, 10, 20 and 40 μM) of Lie for 5 days, respectively. (**B**) Immunoblotting for uncoupling protein 1 (UCP1) and mitochondrial oxidative phosphorylation complex I, II, III, IV and V (COX I–V) in the control (0 μM Rosi) and beige adipocytes. (**C**) After Ros withdrawal, immunoblotting for UCP1 and COX I-V at indicated time points (day 0–5)**.** (**D**) Oxygen consumption rate (OCR) in the control, beige adipocytes and beige adipocytes at day 5 following Rosi withdrawal. The mitochondrial complex inhibitors were injected sequentially in the following order: oligomycin (1 μM), carbonyl cyanide 4 (trifluoromethoxy) phenylhydrazone (FCCP) (0.75 μM), antimycin/rotenone (1 μM each). (**E**) Effects of Lie on the viability of beige adipocytes. Beige adipocytes were exposed to various concentrations (0, 10, 20, 40 and 60μM) of Lie for 5 days, respectively. (**F**) Immunoblotting for UCP1 and COX I–V in beige adipocytes exposed to various concentrations (0, 10, 20 and 40 μM) of Lie for 5 days after Rosi withdrawal, respectively. (**G**) OCR in beige adipocytes exposed to various concentrations (0, 10 and 40 μM) of Lie for 5 days after Rosi withdrawal, respectively. Values shown are means ± standard deviation (SD), # *p* < 0.05 vs. 0 μM Rosi group; * *p* < 0.05 vs. 0 μM Lie group.

3T3-L1 preadipocytes were differentiated into white adipocytes. Browning white adipocytes were induced by treating white adipocytes with 10 μM Rosi for 2 days and we defined them as beige adipocytes in the following text (Figure 1A). As shown in Figure 1B, Rosi significantly upregulated the expression of brown-fat-specific proteins UCP1 and COX I–V. However, the expression of UCP1 and COX I–V were gradually downregulated after Rosi withdrawal (Figure 1C). We further investigated OCR in the control, beige adipocytes and beige adipocytes at day five after Rosi withdrawal (Figure 1D). As revealed in Figure S1A and Figure S1B, compared with the control group, the basal OCR and the maximal OCR were significantly increased by Rosi stimulation (*p* < 0.05). However, this effect was decreased after 5 days Rosi withdrawal. It suggested that mitochondria mass was decreased after Rosi withdrawal, indicating that beige adipocytes reconverted to white adipocytes. 

To understand the potential roles of Lie in prolonging the maintenance of beige adipocytes, we first measured the effect of Lie on cell viability. The cytotoxic effect of Lie on beige adipocytes was evaluated with CCK8 assays after treatment for 5 days. As shown in Figure 1E, cell viability was significantly reduced after treatment with 60 μM Lie. Thus, 10, 20 and 40 μM of Lie were chosen for the following studies. As shown in Figure 1F, compared with control group, Lie treatment retained the level of UCP1 and COX I–V on day five. Additionally, 10 and 40 μM of Lie significantly retained their levels of the basal OCR and the maximal OCR (*p* < 0.05) (Figure 1G, Figure S1C and Figure S1D). Overall, these data strongly demonstrated that Lie inhibited the beige adipocytes recovering to white adipocytes and retained their characteristics after Rosi withdrawal in vitro.

### 3.2. Lie Blocked Mitophagy after Rosi Withdrawal In Vitro

**Figure 2:**
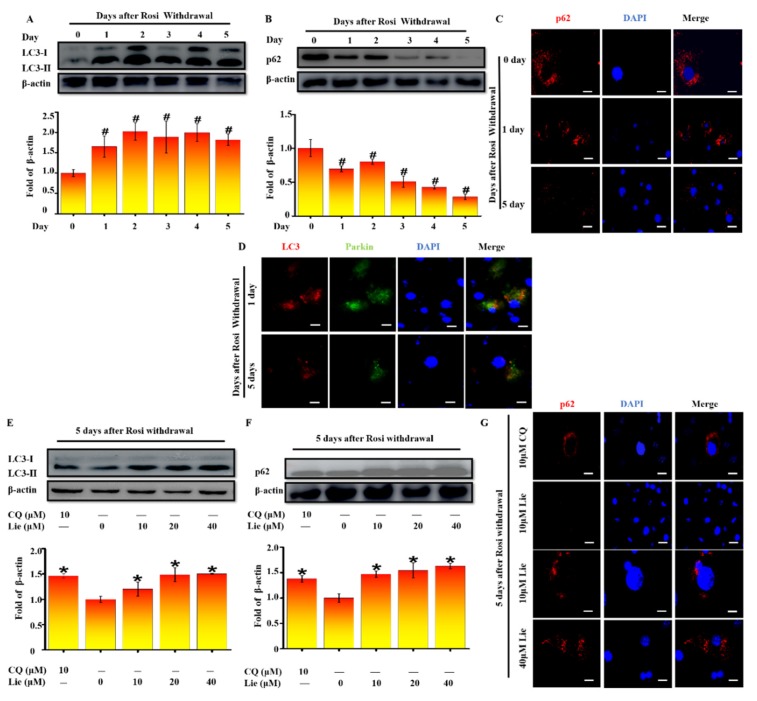
Lie blocked mitophagy after Rosi withdrawal in vitro. After Rosi withdrawal, immunoblotting for microtubule-associated protein 1 light chain 3 (LC3) at indicated time points (day 0–5) (**A**) A densitometric assay of each band. Fold change in optical density relative to day zero in the below of panel of A. After Rosi withdrawal, immunoblotting for selective autophagy adaptor protein (p62) at indicated time points (day 0–5) (**B**) And densitometric assay of each band. Fold change in optical density relative to day zero in the below of panel of **B**. After Rosi withdrawal, beige adipocytes stained with p62 antibodies at indicated time points (**C**) Scale bars, 20 mm. After Rosi withdrawal, beige adipocytes stained with LC3 and Parkin antibodies at indicated time points (**D**) Scale bars, 20 mm. After Rosi withdrawal, beige adipocytes were treated Lie (0, 10, 20 and 40 μM) or chloroquine (CQ, 10μM) for 5 days. Western blot analysis showing the levels of LC3 (**E**) A densitometric assay of each band. Fold change in optical density relative to control (0 μM Lie) in the below of panel of **E**. Western blot analysis showing the levels of p62 (**F**) A densitometric assay of each band. Fold change in optical density relative to control in the below of panel of **F**. Immunoblotting showing the of the levels of p62 (**G**) Scale bars, 20 mm. Values shown are means ± standard deviation (SD), # *p* < 0.05 vs. 0 day after Rosi withdrawal; * *p* < 0.05 vs. 0 μM Lie group.

Increasing evidence indicates that mitophagy promotes the degradation of mitochondria in vivo [38]. To validate whether mitophagy occurred after Rosi withdrawal in vitro, LC3 was firstly detected. As shown in Figure 2A, western blotting results showed that LC3-II/β-actin accumulation was mostly increased after Rosi withdrawal (*p* < 0.05). Two possibilities would explain the LC3-II/β-actin increase: autophagic flux was either inhibited or promoted. As p62 protein serves as a link between LC3 and ubiquitinated substrates, we next assessed levels of p62. On the contrary, the amount of p62 was significantly decreased (*p* < 0.05) (Figure 2B). Moreover, the immunostaining images confirmed the downregulation of p62 from day zero to day five after Rosi withdrawal (Figure 2C). Collectively these data provide evidence that autophagic flux was increased after Rosi withdrawal. Given that Parkin-ubiquitylated mitochondria is necessary for the combination between mitochondrion and autophagosome, whether Parkin was localized at the site of autophagosome was detected. We performed a double staining immunofluorescence of LC3, as autophagosome marker, and Parkin as mitochondria marker. Figure 2D clearly showed that LC3 colocalized with Parkin at day zero and day five after Rosi withdrawal, suggesting that Parkin-mediated mitophagy occurred. Taken together, all results suggested that the beige-to-white adipocytes transition was coupled to an increase in mitophagy.

**Figure 3:**
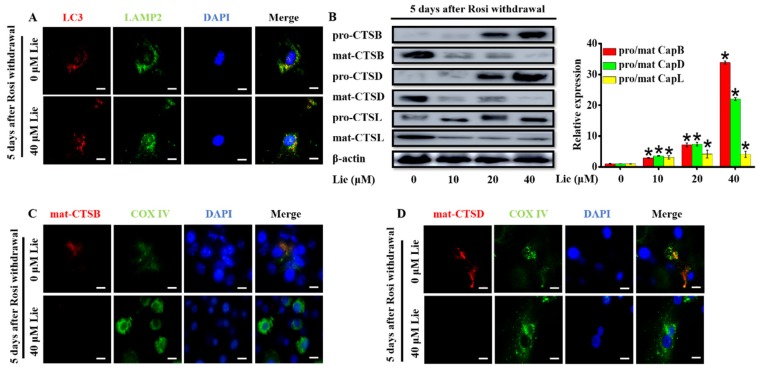
Lie blocked mitophagy flux by impairing the function of lysosomal proteases after Rosi withdrawal in vitro. After Rosi withdrawal, beige adipocytes were subjected to Lie for 5 days. (**A**) Fusion between autophagosomes (anti-LC3) and lysosomes (anti- lysosome-associated membrane protein 2) (anti-LAMP2) was evident in Lie (0 and 40 µM)-treated beige adipocytes (yellow in merged images). Scale bars, 20 mm. Western blot analysis showing the levels of pro-cathepsin B (pro-CTSB), mature-cathepsin B (mat-CTSB), pro-cathepsin D (pro-CTSD), mature-cathepsin D (mat-CTSD), pro-cathepsin L (pro-CTSL) and mature-cathepsin L (mat-CTSL) (**B**) A densitometric assay of each band. Fold change in optical density relative to control group (0 μM Lie) in the right of panel of B. (**C** and **D**) After Rosi withdrawal, beige adipocytes were treated with Lie (0 and 40 µM) for 5 days, which was prepared and subjected to immunostaining with anti-CTSB/anti-CTSD and anti-COX IV. Scale bars, 20 mm. Values shown are means ± standard deviation (SD), * *p* < 0.05 vs. 0 μM group.

Many studies showed that blocking mitophagy flux could retain the characteristics of beige adipocytes [24]. Thus, we explored whether Lie could block mitophagy flux after Rosi withdrawal. As shown in Figure 2E, Lie significantly enhanced the LC3-II/β-actin expression level, showing the autophagosome accumulation (*p* < 0.05). In addition, we found Lie significantly upregulated p62 expression (*p* < 0.05), indicating that the mitophagy flux was blocked (Figure 2F). The immunostaining images in Figure 3G further confirmed this. What is more, we found Lie treatment showed nearly the same effect as chloroquine (CQ) (an autophagy inhibitor as a positive control) did. Taken together, all data strongly suggested that Lie could block mitophagy during the beige-to-white transition after Rosi withdrawal in vitro.

### 3.3. Lie Blocked Mitophagy Flux by Impairing the Function of Lysosomal Proteases after Rosi Withdrawal In Vitro 

A probable explanation for the impaired mitophagy flux could be an inhibition of autophagosomes fusion with lysosomes [39]. To determine whether Lie inhibited mitophagosome fusion with lysosomes, we investigated the colocalization of LC3, an autophagosome marker and LAMP2, a marker for lysosomal membranes, by a double staining immunofluorescence. As shown in Figure 3A, Lie did not cause any changes in the LC3-LAMP2 colocalization, meaning that Lie did not block the autophagosomes fusion with lysosomes. Thus, we speculated if Lie influenced the function of the lysosomal. So, we further examined the effects of Lie on lysosomal function.

In the process of mitochondria degradation, cathepsins containing cathepsin B (CTSB), cathepsin D (CTSD) and cathepsin L (CTSL) are the crucial lysosomal proteases [40,41]. In order to determine whether Lie had an influence on lysosome proteases, the expression levels of lysosomal proteases after treatment with Lie for 5 days were evaluated (Figure 3A). Western blotting results showed Lie dose-dependently promoted the accumulation of pro-CTSB, pro-CTSD and pro-CTSL, suggesting that Lie impaired mitophagy flux by de-maturing the formation of lysosomal cathepsin (*p* < 0.05) (Figure 3B). In addition, we performed a double staining immunofluorescence of cathepsin B/D, and COX IV, as a mitochondrial marker. Figure 3C,D clearly showed that cathepsin B and D did not colocalize with COX IV, suggesting that mitochondria were not being degraded by cathepsin B and D in Lie group. Overall, these results showed that Lie inhibited lysosomal cathepsin activity by promoting the accumulation of pro-CTSB, pro-CTSD and pro-CTSL, which subsequently inhibited mitophagy flux, ultimately preventing mitochondria degradation.

### 3.4. Lie Retained the Molecular Characteristics of Beige Adipocytes after β3-AR Agonist Withdrawal In Vivo 

**Figure 4:**
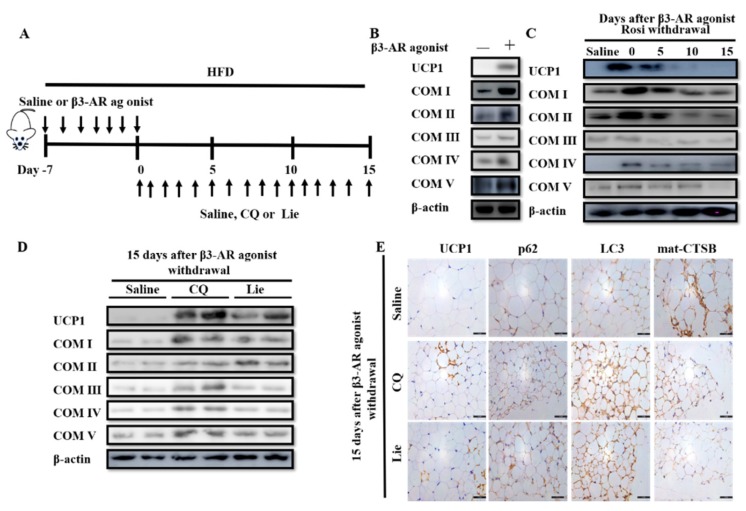
Lie retained the molecular characteristics of beige adipocytes after β3-AR agonist withdrawal in vivo. (**A**) Schematic illustration about in vivo experiments. Mice were treated with the HFD+β3-AR agonist or high fat diet (HFD+) saline for 7 days consecutively and after β3-AR agonist withdrawal, the mice were treated with HFD + 60 mg·kg^−1^ CQ, HFD + 60 mg·kg^−1^ Lie or HFD + saline (control), respectively. (**B**) Immunoblotting for uncoupling protein 1 (UCP1) and mitochondrial oxidative phosphorylation complex I–V (COX I–V) in the inguinal white adipocyte tissue (WAT) depots of mice treated with the HFD+β3-AR agonist or HFD + saline for 7 days consecutively. (**C**) Immunoblotting for UCP1 and COX I–V in the inguinal WAT depots of mice at indicated time points after β3-AR agonist withdrawal. (**D**) Mice were treated with the HFD+β3-AR agonist for 7 days consecutively and after β3-AR agonist withdrawal, the mice were treated with HFD + 60 mg·kg^−1^ CQ, HFD + 60 mg·kg^−1^ Lie or HFD + saline (control), respectively. Inguinal WAT depots were harvested for immunoblotting analyses and immunohistochemical analysis. (**D**) Immunoblotting analyses showing that the levels of UCP1 and COX I–V. (**E**) Immunohistochemical analysis for UCP1, p62, LC3 and mat-CTSB.

In order to obtain beige adipocytes in fat depots, the mice were intraperitoneally injected with β3-AR agonist for 7 days as reported previously [42]. In the following 15 days, beige adipocytes were either treated with saline, 60 mg·kg^−1^ CQ or 60 mg·kg^−1^ Lie, respectively (Figure 4A). β3-AR agonist treatment increased the expression levels of UCP1 and COX I–V (Figure 4B). However, after β3-AR agonist withdrawal, the expression levels of UCP1 and COX I–V were gradually decreased (Figure 4C). These data indicated that the beige-to-white adipocyte transition was accompanied with mitochondria mass decreasing in vivo. More interestingly, treatment with Lie or CQ for 15 days retained the high levels of UCP1 and COX I–V after β3-AR agonist withdrawal (Figure 4D), suggesting that Lie retained the molecular characteristics of beige adipocytes. Further, either Lie or CQ treatment alone caused an increase in the number of UCP1-positive, p62-positive, and LC3-positive cells (brown color) when compared with the saline group (Figure 4E). On the contrary, Lie dramatically decreased the number of mat-CTSB positive cells. These data were consistent with the results from the in vitro experiments. All data indicated that Lie retained the molecular characteristics of beige adipocytes by inhibiting lysosomal proteases-mediated mitophagy. 

### 3.5. Lie retained the Functional Characteristics of Beige Adipocytes and Ameliorated HFD-Induced Obesity In Vivo 

**Figure 5:**
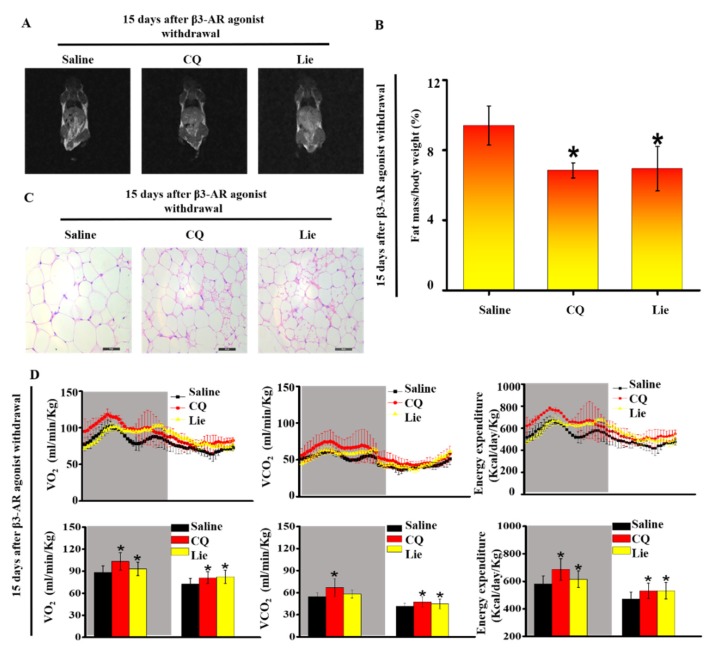
Lie retained the functional characteristics of beige adipocytes and ameliorated HFD-induced obesity in vivo. Mice were treated with the HFD + β3-AR agonist for 7 days consecutively and after β3-AR agonist withdrawal, the mice were treated with HFD + 60 mg·kg^−1^ CQ, HFD + 60 mg·kg^−1^ Lie or HFD + saline (control), respectively. Magnetic resonance imaging (MRI) images showing fat distribution in mice (**A**) Fat mass content (*n* = 3/group) (**B**) Inguinal WAT depots were harvested for H&E images (**C**) Quantification of whole-body O_2_ consumption, CO_2_ production and energy expenditure of mice treated with HFD + 60 mg·kg^−1^ CQ, HFD + 60 mg·kg^−1^ Lie or HFD + saline for 15 days after β3-AR agonist withdrawal. O_2_ consumption, CO_2_ production and energy expenditure were measured during a 12 h light-dark cycle (**D**) *n* = 3/group. Values shown are means ± standard deviation (SD), * *p* < 0.05 vs. saline group.

Magnetic resonance images and H&E staining of the inguinal adipocytes showed that CQ and Lie alone significantly decreased the size of fat depots after withdrawal β3-AR agonist (Figure 5A). The administration with CQ and Lie could suppress the rate of body weight gain (*p* < 0.05) (Table 1). Compared with saline group, treatment with either CQ or Lie had no significant change in TC, TG, LDL-C, lean content and water content (Table 1), but lowered the HDL-C level (*p* < 0.05) (Table 1). The fat content was highest in the saline group whereas Lie group showed the lowest fat content, close to that of CQ group (Figure 5A and 5B) (*p* < 0.05). The adipocyte size of Lie and CQ groups were smaller than those of saline group (Figure 5C). The size of the lipid droplet has a converse relation with energy expenditure [10]. The size of the lipid droplet decreases, supporting the notion that CQ or Lie could enhance energy expenditure (Figure 5B). Importantly, metabolic assessment showed an increase in VO_2_ consumption, VCO_2_ production and energy expenditure in response to CQ and Lie without a change in spontaneous activity (Figure S2A). These results indicated that Lie retained the functional characteristics of beige adipocytes and ameliorated HFD-induced obesity. 

## 4. Discussion

Lie, a major isoquinoline alkaloid, extracted from the seed embryo of *Nelumbo nucifera Gaertn*, has been used as an autophagy inhibitor for treating breast cancer [31]. Interestingly, recent studies reveal that inhibiting mitophagy can maintain beige adipocytes characteristics in vivo [22,38]. Owing to these researches, preclinical trials evaluating the utility of mitophagy inhibitors for treating obesity have been initiated [3]. In this study, we firstly demonstrated that Lie could retained the molecular characteristics of beige adipocytes to relieve obesity by inhibiting mitophagy. 

Nowadays, the association between Rosi and white-to-beige adipocyte transition has been clarified. Rosi, as a PPARγ agonist, induces mitochondrial biogenesis and makes white adipocytes turn to beige adipocytes [43]. However, the change of beige adipocytes after Rosi withdrawal has been unclear. Here, our results showed that after Rosi withdrawal, mitophagy-mediated mitochondria clearance occurred. More interestingly, Lie retained the molecular characteristics of beige adipocytes after Rosi withdrawal in vitro.

Mitophagy inhibition has been recently regarded as an effective method to retain the characteristics of the beige adipocyte [22]. Mitophagy is a fundamental dynamic process. First, mitochondria are sequestered in autophagosomes. Then, autophagosomes fuse with lysosomes to form autolysosomes. Last, the autophagic cargo is degraded by lysosomal proteases in autolysosomes. The efficiency of lysosomal proteases (CTSB, CTSD and CTSL) degradation determines autophagic flux [44,45,46]. CTSB has a great influence on autophagy process and the up-regulation of CTSB has a negative effect on the activation of autophagy in adipocytes [44,47]. CTSD also has an essential role in the process of UCP1 degradation and mitochondria degradation [16,41]. Inhibiting the activity of CTSL could promote autophagy and decrease accumulation of p62 [48]. However, the effect of Lie on mitophagy has been unclear in beige adipocytes. Here, our data showed that Lie induced autophagosome and did not have an influence on autophagosomes-lysosome fusion. But, through down-regulating mat-CTSB, mat-CTSD and mat-CTSL, Lie blocked the degradation of mitochondria in beige adipocytes after Rosi withdrawal.

Prolonged maintenance of beige adipocytes is sufficient to protect mice from diet-induced obesity [22]. Through suppressing autophagy, raspberry ketone (160 mg·kg^−1^, once a day, 8 weeks) can prolong the period of beige adipocytes, thereby reducing the body weight gain and the size of white adipocytes [49]. Adipose-pecific deletion of autophagy-related gene 7 in mice inhibited autophagy and increased the proportion of brown adipose tissue [50]. Parkin KO mice showed higher energy expenditure than wild mice, in part, prolonging retention of beige adipocytes [38,51]. Our results showed that Lie retained the expression levels of UCP1 and COX I–V to 15 days after β3-AR agonist withdrawal. In addition, energy expenditure improved, and body weight gain rate decreased. These data suggested that Lie might be effective in alleviating HFD-induced obesity through prolonging maintenance of beige adipocyte. 

Previous research shows that Rosi can stimulate mitochondria biogenesis and enhance UCP1 function, thereby increasing energy dissipation [43]. To the best of our knowledge, this is the first study to point out that mitophagy occurred after Rosi withdrawal in beige adipocytes. However, the molecular signaling and mechanism that mediates the mitochondria biogenesis-to-clearance after Rosi withdrawal need to be further investigated. 

Natural compounds from alkaloids including matrine, oxymatrine, gramine, cepharanthine and dauricine have been found to possess biological activities, such as anti-hypertension, anti-cancer, anti-inflammations, etc. [52,53]. Previous studies about Lie are mainly focused on anti-cancer, anti-arrhythmias and anti-oxidation [31,54]. However, there is no study about the effect of Lie on treating obesity. In our study, the results first showed that Lie blocked mitophagy flux, ultimately inhibited the beige adipocytes recovering to white adipocytes, and maintained the characteristics of beige adipocyte after stimuli withdrawal. By inhibiting the expression of mat-CTSB, mat-CTSD and mat-CTSL, Lie blocked mitophagy flux. But, the detailed mechanism of Lie acting on lysosomal cathepsins should be clarified in the future. 

In conclusion, our results provided clear evidences that Lie could retain the characteristics of beige adipocytes after stimuli withdrawal in vitro an in vivo. Lie inhibited lysosomal cathepsin activity by promoting the accumulation of pro-CTSB, pro-CTSD and pro-CTSL, which subsequently inhibited mitophagy flux, ultimately retaining the characteristics of beige adipocytes. To the best of our knowledge, our study is the first to suggest that targeting mitophagy inhibition through decreasing lysosomal proteases levels could be an effective treatment for obesity. Lie, as a newly developed natural mitophagy inhibitor, has potential for preventing and treating obesity, which shows a promising future in both research and clinical settings. Pharmacologically blocking mitophagy might also be a novel approach for us to seek more effective bioactive compounds to prevent or treat obesity.

## Figures and Tables

**Table 1:** Metabolic characteristics of study mice treated with HFD + 60 mg kg^−1^ CQ, HFD + 60 mg kg^−1^ Lie or HFD + saline for 15 days after β3-AR agonist withdrawal.

Group	Saline	60 mg·kg^−1^ CQ	60 mg·kg^−1^ Lie
Weight gain rate (%) Lean content (%) Water content (%) TC (mg·dL^−1^) TG (mg·dL^−1^) LDL-C (mg·dL^−1^) HDL-C (mg·dL^−1^)	10.70% ± 2.29% 45.64% ± 0.79% 5.16% ± 1.73% 4.82 ± 0.50 0.92 ± 0.24 0.36 ± 0.07 3.83 ± 0.17	−4.12% ± 0.92% * 45.56% ± 2.03% 4.27% ± 4.27% 4.38 ± 0.13 1.01 ± 0.06 0.38 ± 0.07 3.20 ± 0.07 *	2.71% ± 0.89% * 46.68% ± 2.80% 4.67% ± 0.52% 4.4 ± 0.65 0.88 ± 0.06 0.45 ± 0.32 3.19 ± 0.13 *

TC, Serum total cholesterol; TG, Serum triglycerides; LDL-C, Serum low-density-lipoprotein cholesterol; HDL-C, Serum high-density-lipoprotein cholesterol. * indicates a significant difference from the saline group. * *p* < 0.05 vs. saline group.

## Supplementary Materials

The following are available online at https://www.mdpi.com/2072-6643/11/7/1640/s1, Figure S1: The basal OCR and the maximal OCR in the control, beige adipocytes and beige adipocytes at day 5 following Rosi withdrawal. The basal OCR and the maximal OCR in Beige adipocytes exposed to various concentrations (0, 10 and 40 μM) of Lie for 5 days after Rosi withdrawal. Figure S2: Spontaneous activity during a 12 h light-dark cycle was measured by CLAMS.

## Abbreviations

COX I–V	mitochondrial oxidative phosphorylation complex I: II: III, IV and V
CQ	chloroquine
HDL-C	Serum high-density-lipoprotein cholesterol
HFD	high fat diet
LC3	microtubule-associated protein 1 light chain 3
LDL-C	Serum low-density-lipoprotein cholesterol
Lie	Liensinine
mat-CTSB	mature-cathepsin B
mat-CTSD	mature-cathepsin D
mat-CTSL	mature-cathepsin L
OCR	oxygen consumption rate
OXPHOS	oxidative phosphorylation
pro-CTSB	pro-cathepsin B
pro-CTSD	pro-cathepsin D
pro-CTSL	pro-cathepsin L
Parkin	an E3 ubiquitin ligase that ubiquitinates outer mitochondrial proteins
p62	selective autophagy adaptor protein
Rosi	rosiglitazone
UCP1	uncoupling protein 1
TC	Serum total cholesterol
TG	Serum triglycerides
