# Supplementary material for: Liensinine Inhibits Beige Adipocytes Recovering to white Adipocytes through Blocking Mitophagy Flux In Vitro and In Vivo
Source: Nutrients. 2019 Jul 18;11(7):1640. doi: 10.3390/nu11071640 (PMC6682930; doi:10.3390/nu11071640)
Supplement: Supplementary file 1 [file nutrients-11-01640-s001.pdf]

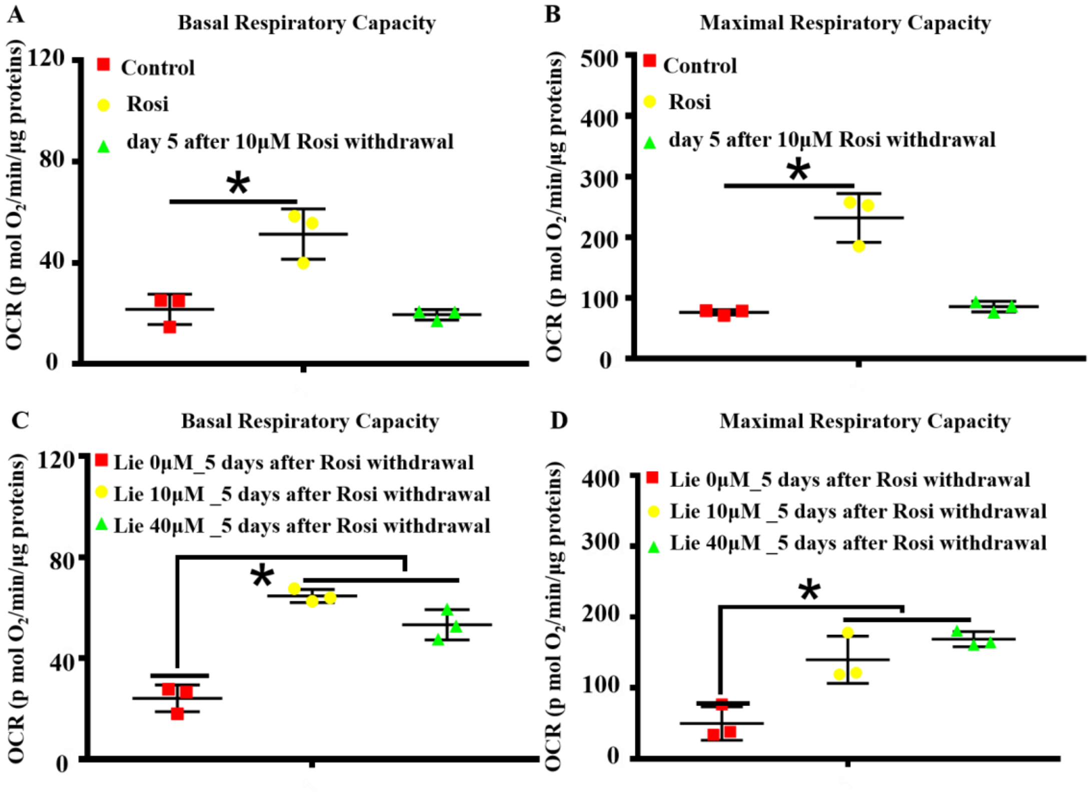

Figure S1. The basal oxygen consumption rate (OCR) (**A**) and the maximal OCR (**B**) in the control, beige adipocytes and beige adipocytes at day 5 following Rosi withdrawal. The basal OCR (**C**) and the maximal OCR (**D**) in Beige adipocytes exposed to various concentrations (0, 10 and 40 μM) of Lie for 5 days after Rosi withdrawal. The mitochondrial complex inhibitors were injected sequentially in the following order: oligomycin (1 μM), carbonyl cyanide p-trifluoromethoxyphenylhydrazone (FCCP) (0.75 μM), antimycin/rotenone (1 μM each).

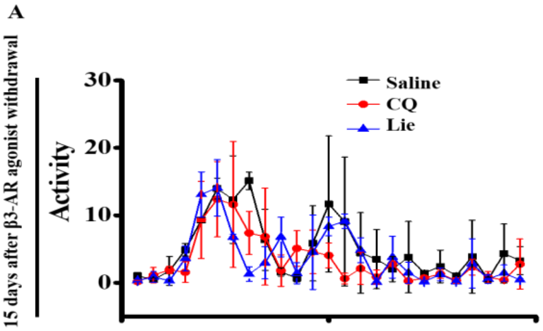

Figure S2. Spontaneous activity during a 12 h light-dark cycle was measured by Comprehensive Laboratory Animal Monitoring System (CLAMS). n=3/group Values shown are means ± standard deviation (SD).
